# Piglet innate immune response to *Streptococcus suis* colonization is modulated by the virulence of the strain

**DOI:** 10.1186/s13567-021-01013-w

**Published:** 2021-12-19

**Authors:** Carlos Neila-Ibáñez, Louise Brogaard, Lola Pailler-García, Jorge Martínez, Joaquim Segalés, Mariela Segura, Peter M. H. Heegaard, Virginia Aragon

**Affiliations:** 1 IRTA, Centre de Recerca en Sanitat Animal (CReSA, IRTA-UAB), Campus de la Universitat Autònoma de Barcelona, 08193 Bellaterra, Spain; 2 OIE Collaborating Centre for the Research and Control of Emerging and Re-Emerging Swine Diseases in Europe (IRTA-CReSA), Bellaterra, Barcelona Spain; 3 Section for Protein Science and Biotherapeutics, DTU Bioengineering, Technical University of Denmark, Kongens Lyngby, Denmark; 4 UAB, Centre de Recerca en Sanitat Animal (CReSA, IRTA-UAB), Campus de la Universitat Autònoma de Barcelona, 08193 Bellaterra, Spain; 5 Departament de Sanitat I Anatomia Animals, Facultat de Veterinària, UAB, 08193 Bellaterra, Barcelona, Spain; 6 Research Group On Infectious Diseases in Production Animals and Swine and Poultry Infectious Diseases Research Centre, Faculty of Veterinary Medicine, University of Montreal, St-Hyacinthe, QC J2S 2M2 Canada; 7 Innate Immunology Group, Center for Diagnostics, DTU Health Tech, Technical University of Denmark, Kongens Lyngby, Denmark; 8 Current Affiliation: Section for Animal Genetics, Bioinformatics and Breeding, Department of Veterinary and Animal Sciences, Faculty of Health and Medical Sciences, University of Copenhagen, Frederiksberg, Denmark

**Keywords:** *Streptococcus suis*, colonization, innate immunity, pig immunity, gene expression, bacterial virulence

## Abstract

*Streptococcus suis* is a zoonotic pathogen of swine involved in arthritis, polyserositis, and meningitis. Colonization of piglets by *S. suis* is very common and occurs early in life. The clinical outcome of infection is influenced by the virulence of the *S. suis* strains and the immunity of the animals. Here, the role of innate immunity was studied in cesarean-derived colostrum-deprived piglets inoculated intranasally with either virulent *S. suis* strain 10 (S10) or non-virulent *S. suis* strain T15. Colonization of the inoculated piglets was confirmed at the end of the study by PCR and immunohistochemistry. Fever (≥40.5 °C) was more prevalent in piglets inoculated with S10 compared to T15 at 4 h after inoculation. During the 3 days of monitoring, no other major clinical signs were detected. Accordingly, only small changes in transcription of genes associated with the antibacterial innate immune response were observed at systemic sites, with S10 inducing an earlier response than T15 in blood. Local inflammatory response to the inoculation, evaluated by transcriptional analysis of selected genes in nasal swabs, was more sustained in piglets inoculated with the virulent S10, as demonstrated by transcription of inflammation-related genes, such as *IL1B*, *IL1A*, and *IRF7*. In contrast, most of the gene expression changes in trachea, lungs, and associated lymph nodes were observed in response to the non-virulent T15 strain. Thus, *S. suis* colonization in the absence of systemic infection induces an innate immune response in piglets that appears to be related to the virulence potential of the colonizing strain.

**Supplementary Information:**

The online version contains supplementary material available at 10.1186/s13567-021-01013-w.

## Introduction

*Streptococcus suis* is a major bacterial pathogen of swine, involved in meningitis, arthritis, septicemia, and acute death, among other clinical syndromes. Disease caused by *S. suis* is more prevalent in nursery pigs, but sucklers and young fatteners can also be affected [1]. Additionally, *S. suis* is a zoonotic agent that is receiving increased scientific interest due to Chinese outbreaks in humans in 1998 and 2005 [2].

*Streptococcus suis* is an early colonizer of the swine upper respiratory tract, mainly found in tonsil and nasal cavity [3]. Newborn piglets experience the first contact with the bacterium in the birth canal during parturition, as *S*. *suis* colonizes the sow’s vaginal tract [4]. In addition, animals housed in the same pen are exposed to horizontal colonization by direct contact or aerosol, especially during outbreaks when animals may shed bacteria in high numbers [5].

*Streptococcus suis* strains are heterogeneous both with respect to antigenicity and virulence [6]. Presently, 29 confirmed serotypes have been described based on the antigenicity of capsular polysaccharides [7, 8]. Pigs are usually colonized by more than one serotype, but only a few strains can induce disease. Serotype 2 is the most frequently isolated serotype from affected organs in diseased individuals (swine and humans) in most parts of the world [9]. However, different virulence results have been reported for the same *S. suis* serotype, or even the same *S. suis* strain [10, 11].

Different animal models have been used to study *S. suis* pathogenesis, including pigs, mice, rabbits, and zebrafish [12]. Clinical disease has been reproduced by respiratory, intraperitoneal, and intravenous routes in pigs, but reproduction of the disease is difficult [12]. After colonization, the development of disease depends on the virulence potential of the strain and the interplay between the host response and the bacteria [13]. Severe disease is caused by excessive inflammation [14], and in vitro studies have demonstrated strong induction of pro-inflammatory cytokines and chemokines by *S. suis* serotype 2 [15–18]. A major obstacle to the investigation of *S. suis* disease in vivo is the fact that systemic disease is not easily induced by challenge via the natural oronasal route of infection. In fact, systemic disease by intranasal challenge is not induced or is strongly reduced in the absence of acetic acid pretreatment or prior viral infection [19, 20]. Thus, to reproduce systemic disease, it seems necessary to inoculate *S. suis* either by injection (e.g. intravenous or intraperitoneal) or intranasally after irritation of the mucosa by pre-treatment.

Only few in vivo *S. suis* challenge studies in pigs have focused on characterizing the host immune responses. Genes related to bacterial recognition (*TLR4*, *MYD88*) and inflammatory responses (*IL6*, *CXCL8*, *CCL2*) have been shown to be expressed in lungs of pigs after intranasal *S. suis* challenge (serotype 2, strain 05ZY), and these responses were enhanced by co-infection with influenza A virus (H1N1) [21]. Intravenous challenge of pigs with *S. suis* serotype 2 (strain SC19) induced expression of bacterial pattern recognition receptors (*TLR2*, *CD14*) in the lung, as well as components of the inflammatory response (*IL1B*, *IL6*, *TNF*, *CXCL8*) [22]. Nasal challenge (after acetic acid pre-treatment) with *S. suis* serotype 2 (strain 05ZY) induced a primarily *TLR2*-dependent cytokine response in the spleen [13]. The hepatic response has been investigated in vivo, showing that clinical and subclinical disease after *S. suis* serotype 2 (strain SS02-0119) challenge by subcutaneous inoculation was accompanied by an acute phase response consisting of the acute phase proteins (APPs) serum amyloid A, C-reactive protein, haptoglobin, pig-MAP and Apo A-I [23]. These studies have thus shown that inflammatory responses can be induced in vivo by *S. suis* serotype 2 challenge.

The present study, performed in cesarean-derived colostrum-deprived (CDCD) piglets, describes the host early immune response in blood, nasal mucosa, and various tissues to intranasal inoculation with *S. suis* T15 and S10. These strains belong to serotype 2 and have shown different virulence in pigs, based on the frequency of clinical signs, leukocytosis, and mortality reported in previous animal experiments [24]. Inoculation of the strains was performed without pre-treatment of the mucosa in order to examine the natural response of the host when encountering *S. suis* strains of different virulence. How this early response might affect disease development is also discussed.

## Materials and methods

### Animal study

Animal experimentation was performed in the BSL3 facilities of IRTA-CReSA (Bellaterra, Spain) following good veterinary practices, in accordance with European (Directive 2010/63/EU) and Spanish (Real Decreto 53/2013) regulation. The experimental study was approved by the Ethics Commission in Animal Experimentation of the *Generalitat de Catalunya* (Protocol number 10201). Four pregnant sows were transported to IRTA facilities and housed for two weeks before delivery, which was performed by cesarean section. The genetic background of these piglets was (Duroc × White Large) ♀ × Landrace ♂, a commercial breed. Piglets were fed the milk substitutive Patavie Porc (Oriane-Celtilait) ad libitum during the first 2 days. Afterwards, animals received Neopigg (Provimi Cargill) mixed with the milk or dry after 10–15 days of age. Piglets were treated with colistin (Colimicina SP, SP Veterinaria S.A., Spain) and enrofloxacin (Baytril 0.5%, Bayer Hispania S.L., Spain), both orally, during the first nine days of life. Twenty piglets, housed in the same box, were included in the study. At 25 days of age (3 days before inoculation), blood samples and nasal swabs were taken from all piglets. Piglets were randomly assigned to 5 groups of 4 piglets each for inoculation and euthanasia, and housed in 3 separated boxes, depending on the inoculum assigned to them. One group was inoculated with strain T15 and euthanized 1 day post-inoculation (dpi), while a second group that was also inoculated with strain T15 was euthanized at 3 dpi. Similarly, two groups were inoculated with strain S10 and were euthanized at 1 and 3 dpi, respectively. A fifth group was inoculated with PBS (Phosphate Buffered Saline) and euthanized at 1 dpi, as negative control. On day 28 of life, inoculation was performed intranasally with a nasal atomizer (MAD Nasal™, Teleflex, Athlone, Ireland) with 2 mL of 1.1 × 10^9^ CFU/mL of *S. suis* T15 (non-virulent serotype 2 strain) or with 2 mL of 1.8 × 10^9^ CFU/mL of *S. suis* S10 (virulent serotype 2 strain), while the control group was inoculated with 2 mL of PBS. For the three groups, the inoculated volume was split between the two nostrils. Strains were provided by Dr Astrid de Greeff and Dr Norbert Stockhofe from Wageningen Bioveterinary Research (Wageningen University & Research, the Netherlands). After inoculation, piglets were supervised for clinical signs, including rectal temperature.

To study the innate immune response to the inoculated strains, early time points were chosen for sampling. Nasal swabs and blood were collected 4 h after inoculation, 1, 2 and 3 dpi. After euthanasia, piglets were examined by necropsy and lesion scores were calculated as a combination of the severity of the lesions and the number of body sites affected. In addition, samples from tissues (trachea, cranial and caudal lobes of the lung, submandibular and tracheobronchial lymph nodes, spleen, and liver) were collected. To ensure RNA integrity, blood samples were obtained in PAXgene Blood RNA tubes (Becton Dickinson, Spain) which were kept at room temperature 4 h and subsequently stored at 4 °C for 72 h and ultimately transferred to −20 °C. Nasal swabs and tissues were immediately submerged in RNAlater (Invitrogen, Spain) and stored at 4 °C overnight to allow thorough penetration of the stabilizing solution into the tissue and subsequently stored at −20 °C until RNA extraction was performed.

### Detection of *S. suis* serotype 2 by PCR and immunohistochemistry (IHC)

Additional nasal swabs were taken at necropsy for detection of *S. suis* by PCR. Swabs were resuspended in PBS and DNA was extracted using the Nucleospin Blood kit (Macherey–Nagel, Germany). Four µL of DNA (between 42.0 and 867.2 ng) were used in the PCR to detect the serotype of the challenge strains, serotype 2, as previously described [25].

For IHC, tissue samples from respiratory tract, including nasal turbinates, cribriform plate of ethmoid, trachea, and caudal lung lobe, as well as submandibular and tracheobronchial lymph nodes, were fixed by immersion in 10% buffered formalin and embedded in paraffin. Bacterial antigen detection in tissues was performed by IHC using a rabbit monoclonal anti-*S. suis* serotype 2 antibody (SSI Diagnostica, DK), followed by BrightVision Alkaline Phosphatase (AP)-conjugated anti-rabbit immunoglobulin G (IgG; Immunologic) and Vector Red (Vector Labs). Slides were counter stained with hematoxylin [26]. Additionally, another consecutive slide from each tissue was stained with hematoxylin–eosin to study the lesions.

### RNA extraction and quality control

Extraction of total RNA from lymph nodes, lungs, trachea, spleen, and liver was performed using the miRNeasy Mini Kit (Qiagen) according to the manufacturer’s instructions. Briefly, approximately 30 mg of RNAlater stabilized tissue was homogenized in 1 mL QIAzol Lysis Reagent (in kit) using M-tubes (Miltenyi Biotec) and a gentleMACS Dissociator (Miltenyi Biotec). Total RNA was isolated from the homogenate by column-based extraction, including on-column DNase digestion of contaminating genomic DNA using the RNase-Free DNase Set (Qiagen) according to the manufacturer’s instructions. Total RNA was eluted in 50 µL RNase-free water and stored at −80 °C.

Extraction of total RNA from whole blood collected in PAXgene Blood RNA Tubes was performed using the PAXgene Blood miRNA Kit (Qiagen) according to the manufacturer’s instructions, including on-column DNase digestion as above. RNA was eluted in 40 µL BR5 buffer (in kit) and stored at −80 °C.

Total RNA from RNAlater stabilized nasal swabs was extracted using an in-house optimized protocol. First, the RNAlater containing the swab was mixed with one volume (1 mL) RNA Lysis Buffer from the Quick-RNA Microprep Kit (Zymo Research) and vortexed followed by 5 min incubation at room temperature. Then the swab was removed, and the sample transferred to a 15 mL tube and mixed with 2.5 volumes (5 mL) cold (< 0 °C) 100% ethanol, followed by vortexing and 30 min incubation at −20 °C. The supernatant was carefully removed with a pipette and the precipitate was washed twice with 70% ethanol at room temperature. The precipitate was dissolved in 1 mL RNase-free water and 700 µL was transferred to a Zymo-Spin IC Column (from the Quick-RNA Microprep Kit) and centrifuged at 10000 × *g* for 30 s and flow-through was discarded. This was repeated until the entire sampled had been passed through the column. From this point the Quick-RNA Microprep Kit protocol for RNA purification was followed according to the manufacturer’s instructions, including on-column DNase digestion of contaminating genomic DNA. Total RNA was eluted in 15 µL RNase-free water and stored at −80 °C.

RNA concentration (ng/µL) and purity (A_260_/A_280_ and A_260_/A_230_ ratios) were assessed using a NanoDrop 1000 spectrophotometer (Thermo Scientific). RNA integrity number (RIN) was measured using an Agilent 2100 Bioanalyzer (Agilent Technologies) and the RNA 6000 Nano Kit (Agilent Technologies) (Additional file 1).

### Transcriptional analysis

Two replicates of cDNA were synthesized from each RNA sample using the QuantiTect Reverse Transcription Kit (Qiagen) according to the manufacturer’s instructions employing 500 ng RNA for each synthesis for all tissue and blood RNA samples. For the nasal swabs, due to limited amounts of sample and low RNA yields, cDNA synthesis was performed using as much RNA as was possible for the individual samples, varying from 33 to 323 ng per cDNA synthesis. Two no-reverse transcriptase controls (reaction not containing reverse transcriptase, negative controls) were made for each tissue/sample type.

All cDNA samples (diluted 1:10 in low-EDTA TE buffer) were pre-amplified using the TaqMan PreAmp Master Mix Kit (Applied Biosystems) in combination with a primer mix (each primer at 200 nM) containing all primer pairs to be used in the subsequent qPCR analysis (see below for details on primer design). All cDNA samples from tissues and whole blood were pre-amplified using 18 cycles of amplification, while cDNA samples from nasal swabs were pre-amplified using 22 cycles of pre-amplification. Following pre-amplification, residual primers were digested using Exonuclease I (New England BioLabs). Pre-amplified, exonuclease treated cDNA was diluted 1:10 in low-EDTA TE buffer for use in qPCR, and pools of pre-amplified, exonuclease treated cDNA were prepared from each of the tissue/sample types to produce dilution series in order to experimentally determine qPCR efficiency of all assays (primer pairs) for all investigated tissue/sample types. In addition, a non-template control was prepared to check for background fluorescence build-up of all primer pairs in the absence of cDNA template.

qPCR analysis was carried out using the high-throughput platform BioMark (Fluidigm) using 192.24 Dynamic Array IFC chips (Fluidigm) (192 samples in combination with 24 assays, used for lung tissues, trachea, nasal swabs) or 96.96 Dynamic Array IFC chips (Fluidigm) (96 samples in combination with 96 assays, used for lymph nodes, liver, spleen, blood). All assays (primer pairs) employed in the present study were designed in-house and purchased from Sigma-Aldrich. All qPCR primer sequences and amplification efficiencies can be found in Additional file 2. Whenever possible, primers pairs were designed to span intron/exon borders in order to prevent amplification of potentially contaminating genomic DNA. qPCR was carried out using a sample mix comprising TaqMan Gene Expression Master Mix (Applied Biosystems), DNA Binding Dye (Fluidigm), EvaGreen Dye (Biotium), and pre-amplified, exonuclease treated cDNA (diluted 1:10 in low-EDTA TE buffer). The individual assay mixes consisted of Assay Loading Reagent (Fluidigm) and primer pairs (20 µM for each primer). After loading all samples and reagents onto the chips using appropriate controllers (RX IFC Controller [Fluidigm] for 192.24 Dynamic Array IFC chips and HX IFC Controller [Fluidigm] for 96.96 Dynamic Array IFC chips), chips were transferred to the BioMark instrument for 35 cycles of amplification followed by melting curve analysis to ensure specific amplification.

Amplification curves, melting curves, and standard curves (dilution series) were evaluated using the Fluidigm Real-Time PCR Analysis software (v. 4.1.3). The GenEx software (v. 6) was used to correct C_q_ values with the obtained qPCR efficiencies, to evaluate potential reference genes for data normalization with the geNorm [27] and NormFinder [28] algorithms and subsequently perform normalization, to average technical repeats, and to convert C_q_ values to linear scale by computing relative quantities. Different subsets of reference genes were found appropriate for data normalization in different tissues based on the abovementioned reference gene evaluation: whole blood: *YWHAZ*, *RPL13A*, *HPRT1*; nasal swabs: *B2M*, *RPL13A*, *PPIA*; trachea: *RPL13A*, *GAPDH*, *HPRT1*; lung: *GAPDH*, *HPRT1*, *B2M*, *RPL13A*; submandibular lymph node: *PPIA*, *YWHAZ*, *RPL13A*; tracheobronchial lymph node: *B2M*, *HPRT1*, *PPIA*, *YWHAZ*, *RPL13A*, *ACTB*; liver: *PPIA*, *YWHAZ*, *RPL13A*; spleen: *HPRT1*, *PPIA*, *YWHAZ*.

### Statistical analysis

Data analyses were performed with R (v. 4.0.2,  [29]). Rectal temperature after inoculation was analyzed using ANOVA with Tukey’s Honest Significant Difference (HSD) post-hoc test with interaction between inoculated groups and time points. In order to compare changes in gene expression between groups, relative transcript quantities were calculated; for longitudinal samples (nasal swabs and blood) gene expression levels at 4 h post-inoculation and 1, 2, and 3 dpi were normalized against 3 days before inoculation. Statistical significance of the gene expression changes in whole blood and nasal swab samples was assessed by linear mixed effects regression with interaction between the different time points and the inoculum groups, taking into account animal ID as random-effect. For necropsy samples (all other tissue samples, taken at 1 and 3 dpi), normalization was done against the values of the PBS inoculated group. Statistical significance of the gene expression changes in necropsy tissues was analyzed using ANOVA with Tukey’s HSD post-hoc test with interaction between inoculated groups and time points. A confidence level of 95% was considered as statistically significant (*P* < 0.05).

## Results

### Clinical signs and lesions after inoculation

**Figure 1:**
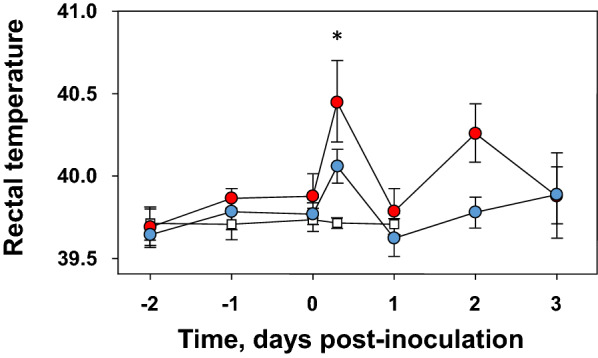
**Rectal temperature before and after** ***S. suis*** **intranasal inoculation**. Mean and standard deviation of rectal temperatures of the piglets before and after intranasal inoculation with *S. suis* strains T15 (non-virulent, blue circles) or S10 (virulent, red circles) (*n* = 8 from -2 to 1 day post-inoculation [dpi]; *n* = 4 at 2 and 3 dpi, for both strains). A group of piglets was inoculated with PBS as control (white squares; *n* = 4 for all time points). * Statistically significant (*P* < 0.05) difference between S10 and PBS groups.

Few clinical signs were observed, comprising mild tremors at 2 and 3 dpi in two piglets inoculated with S10. Differences among the groups were observed in rectal temperature after the inoculation of the two strains. Although both groups of *S. suis* inoculated piglets had higher temperature than the control group (PBS inoculated) at 4 h after the challenge, this difference was statistically significant only in the piglets inoculated with S10 (ANOVA Tukey’s HSD, *P* = 0.044; Figure 1). Furthermore, the number of piglets with rectal temperature higher than 40.5 °C at 4 h post-inoculation was greater in the group inoculated with S10 (5 out of 8) than in the T15 group (1 out of 8); however, no statistical difference was found between the two *S. suis*-inoculated groups. Temperatures at later time points were also recorded, and although S10 gave rise to higher temperature than T15 at 2 dpi, no statistical differences were found (Figure 1).

Gross lesions identified at necropsy were in general mild, affecting animals in all groups, including the PBS challenged group. None of these lesions could be ascribed to the *S. suis* challenge as *S. suis* was not re-isolated or detected by PCR from any of the lesions.

In the histological evaluation, no apparent lesions were found in most of the tissues (98/120) and were not consistent with characteristic *S. suis* pathology, with no differences among the three groups.

### *Streptococcus suis* serotype 2 detection in the respiratory tract after inoculation

**Figure 2:**
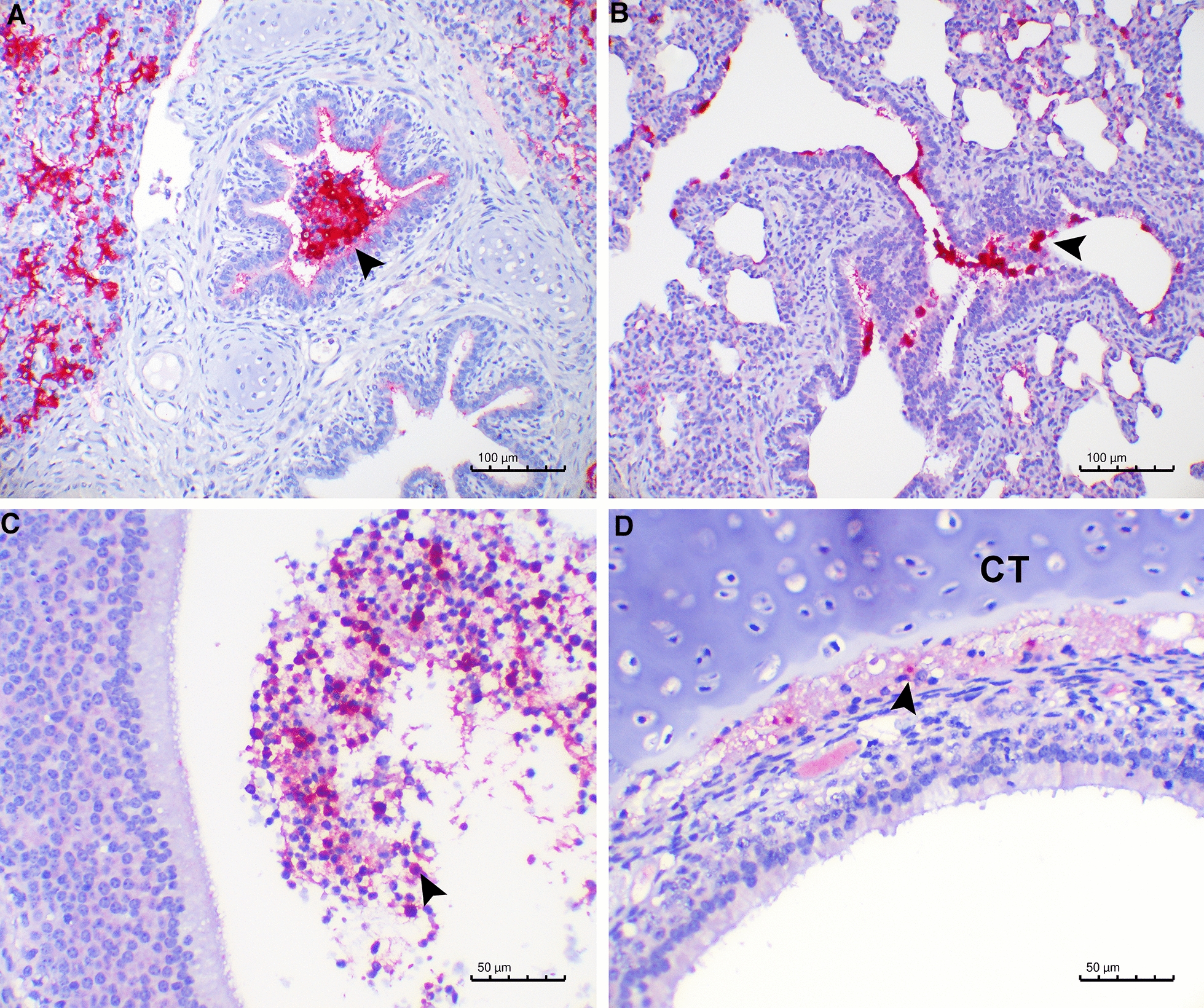
**Detection of** ***S. suis*** **serotype 2 by immunohistochemistry**. Piglets were intranasally inoculated with *S. suis* T15 (non-virulent) or S10 (virulent) strains, and tissues were collected at 1 and 3 days post-inoculation (dpi). A: Bacteria present at 1 dpi in the bronchiole (arrowhead) and alveolar lumen of a piglet inoculated with T15. B: Bacteria present at 1 dpi in the epithelial surface of bronchioles (arrowhead) and alveoli of a piglet inoculated with S10. C: Presence of bacteria at 3 dpi in neutrophils in the mucus of the cribriform plate of ethmoid of a piglet inoculated with T15 (arrowhead). D: Bacteria next to the cartilage of the cribriform plate of ethmoid (CT) at 3 dpi, from a piglet inoculated with S10 (arrowhead).

*S. suis* serotype 2 was detected by PCR in nasal swabs taken postmortem in piglets inoculated with T15 (7/8) or S10 (7/8). Amplification was more intense in nasal swabs from piglets inoculated with S10 than with T15, especially at 3 dpi (three samples from T15 inoculated piglets yielded a weak amplification and one was negative in the PCR, while two samples from S10 inoculated piglets yielded a moderate amplification, one a strong amplification and one was negative in the PCR). Using IHC, *S. suis* serotype 2 was detected in the upper respiratory tract for both strains, mostly in the mucus but also in the epithelium of the nasal cavity (2/8 for T15 and 4/8 for S10), cribriform plates of ethmoid (7/8 for T15 and 8/8 for S10), and tracheas (3/8 for T15 and 1/8 for S10). Immunolabelling was also found in the alveolar lumen of the lungs (4/8 for T15 and 5/8 for S10) (Figures 2A and B), but there was no detection in any of the lymph nodes analyzed. In the cribriform plate of ethmoid, T15 bacteria were found only in the mucus (Figure 2C), while some S10 bacteria were detected deep in the tissue, close to the cartilage (Figure 2D). This latter location was not observed in any of the animals infected with the T15 strain. Thus, *S. suis* serotype 2 was detected in all inoculated animals by either PCR (14/16) or IHC (15/16), but not in the piglets from the non-infected control group.

### Local and systemic transcriptional responses to virulent and non-virulent *S. suis* inoculation

High quality RNA was obtained from lymph nodes, trachea, lungs, spleen, liver, and whole blood. RNA obtained from nasal swabs was of sub-optimal quality, and care was therefore taken when choosing a strategy for transcriptional analysis of these samples. This included limiting the focus to relatively few genes that could be expected to be strongly induced during an inflammatory antibacterial response, as well as assaying the transcription of several of the investigated genes with two independent assays (two different primer pairs targeting the same mRNA transcript at non-overlapping sites). Mean and range of RNA quality for the different tissues are summarized in Additional file 1. Gene expression in longitudinal samples (whole blood and nasal swabs) and in necropsy samples (all other tissues) were compared to the expression at 3 days before challenge and the PBS group, respectively, as indicated above (see section “Materials and methods”).

Generally, only small changes in gene expression were observed in *S. suis*-challenged animals, with the majority of the transcriptional regulation being <2-fold either up- or down-regulated, for both the S10 and T15 strains. In addition, only some of these changes were statistically significant, probably due to the considerable individual variation in gene expression levels observed within the groups of animals.

**Figure 3:**
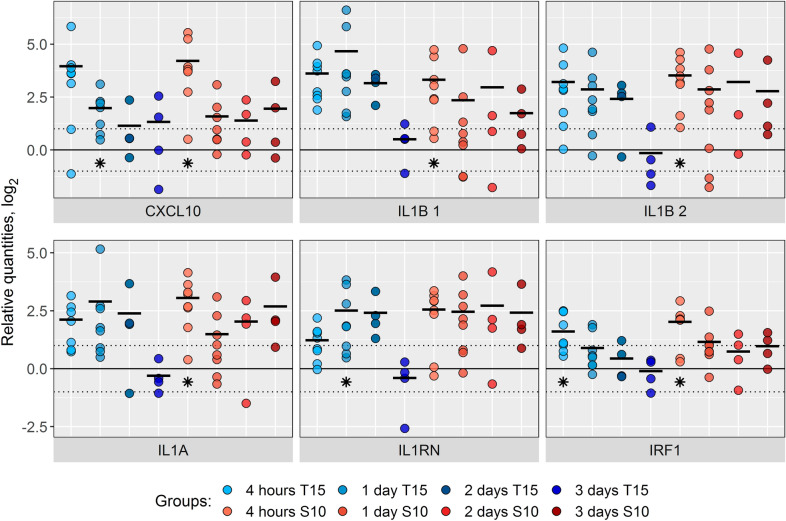
**Relative gene expression in nasal samples after** ***S. suis*** **intranasal inoculation.** Log_2_ of the individual values and mean (black bars) of the relative gene expression in nasal samples in *S. suis* inoculated piglets (gene expression was normalized relative to the 3 days before challenge mean for each inoculated group). Piglets were intranasally inoculated with *S. suis* T15 (non-virulent, blue bars) or S10 (virulent, red bars) strains, and nasal swabs were taken at 4 h and 1, 2 and 3 days after inoculation. Genes showing significant difference (*P* < 0.05) in pairwise analysis when comparing different time points in the *S. suis* inoculated groups or between strains at the same time point, and with a mean greater than 2-fold change (log_2_ = 1) are shown. * *P* < 0.05 when comparing versus their respective 3 days before challenge time point. *n* = 8 for both strains at 4 h post-infection and 1 dpi, *n* = 4 for both strains at 2 and 3 dpi. *IL1B* 1 and *IL1B* 2 are both *IL1B* assays consisting of two different primer pairs targeting non-overlapping sites in the *IL1B* transcript. All expression values and significant differences can be found in Additional file 3.

**Figure 4:**
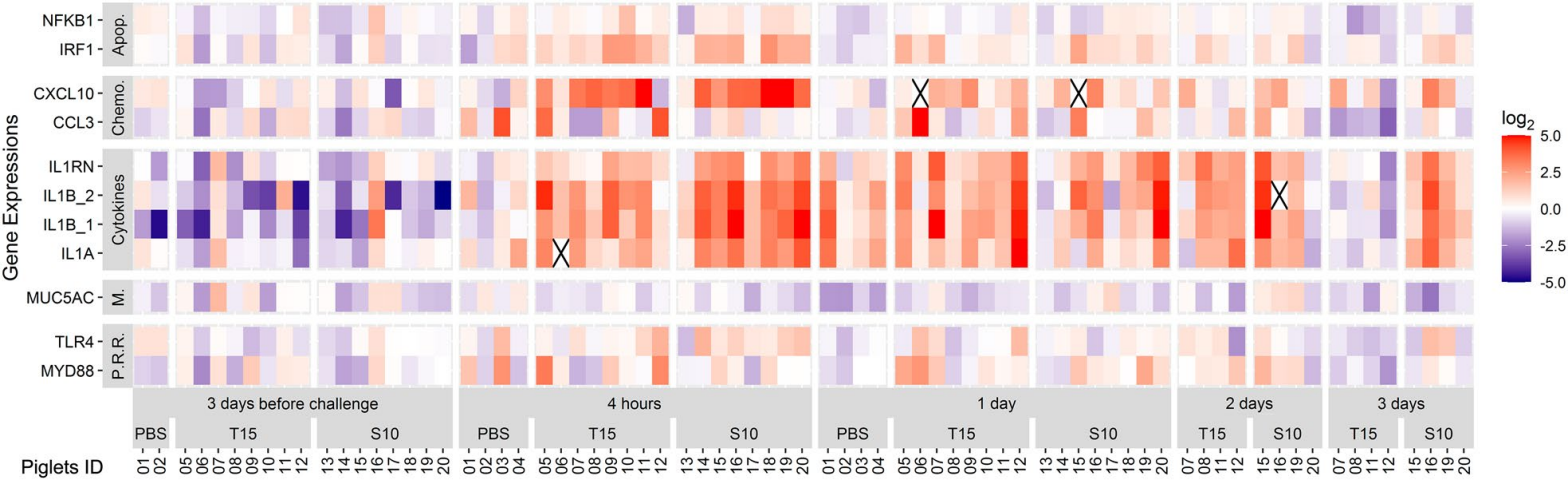
**Gene expression in nasal samples after** ***S. suis*** **inoculation.** Samples were taken at 3 days before, and 4 h, 1, 2, and 3 days after the intranasal inoculation of *S. suis* strains T15 (non-virulent) and S10 (virulent). A group of piglets inoculated with PBS are also shown and served as control. All the genes found to be quantifiable are shown irrespectively of their statistical significance. Gene expression was normalized relative to the 3 days before challenge mean for each inoculated group and log_2_ transformed. Values are presented as a heat map. Numbers in abscissa axis represent animal ID. Color scale was limited to ± 5 and out of bounds values displayed with the maximum intensity color. Gene functional groups: Apop.: Apoptosis; Chemo.: Chemokines; M.: Miscellaneous; P.R.R.: Pattern Recognition Receptors. *IL1B_1* and *IL1B_2* are both *IL1B* assays consisting of two different primer pairs targeting non-overlapping sites in the *IL1B* transcript. Samples marked with a black cross: expression level not quantifiable.

However, a group of genes showed quite pronounced transcriptional responses in nasal swab samples and clearly demonstrated differential host responses after virulent and non-virulent challenge, with more genes consistently up-regulated by S10 at 4 h post-inoculation than by T15 (Figures 3 and 4). These genes included pro- and anti-inflammatory genes *IL1A*, *IL1B*, *IL1RN*, and *IRF1*, as well as the chemokine *CXCL10*, and were induced early in the nasal mucosa after challenge with both strains (Figure 3, Additional file 3). This response seemed to continue unabated throughout the experiment in the piglets inoculated with the virulent S10 strain whereas the response to the T15 challenge was shorter and had a tendency to return to baseline levels by day 3 (Figures 3 and 4). Despite the changes observed between strains (Figures 3 and 4, Additional file 3), specially at 3 dpi, none of these were statistically significant, probably due to the low number of piglets.

**Figure 5:**
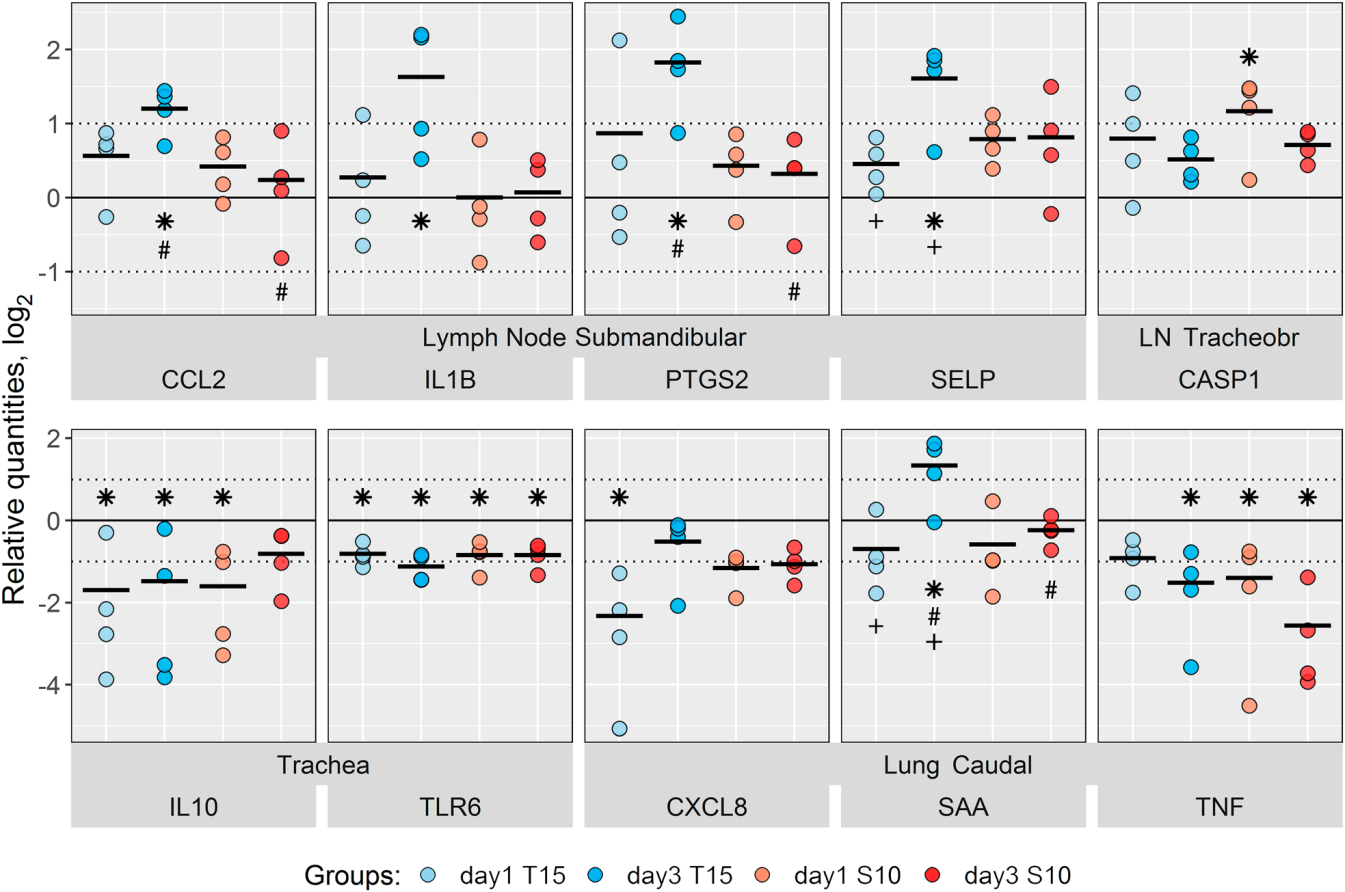
**Relative gene expression in different tissues after** ***S. suis*** **intranasal inoculation.** Log_2_ of the individual values and mean (black bars) of the relative gene expression in different tissues in *S. suis* inoculated piglets (gene expression was normalized relative to the PBS group). Piglets were intranasally inoculated with *S. suis* T15 (non-virulent, blue bars) or S10 (virulent, red bars), and necropsies were performed at 1 and 3 days post-infection. The values and means are shown for the indicated groups (challenge strain and time point) having at least one significant difference when compared to the PBS group and with a mean higher than 2-fold change (log_2_ = 1). LN Tracheobr: Tracheobronchial lymph node. * indicates significant differences (*P* < 0.05) versus the PBS group. Differences between strains at the same time point are labelled with # and differences between time points for the same strain are labelled with +, *P* < 0.05, in both cases. *n* = 4 for each group. All expression values and significant differences can be found in Additional file 5.

**Figure 6:**
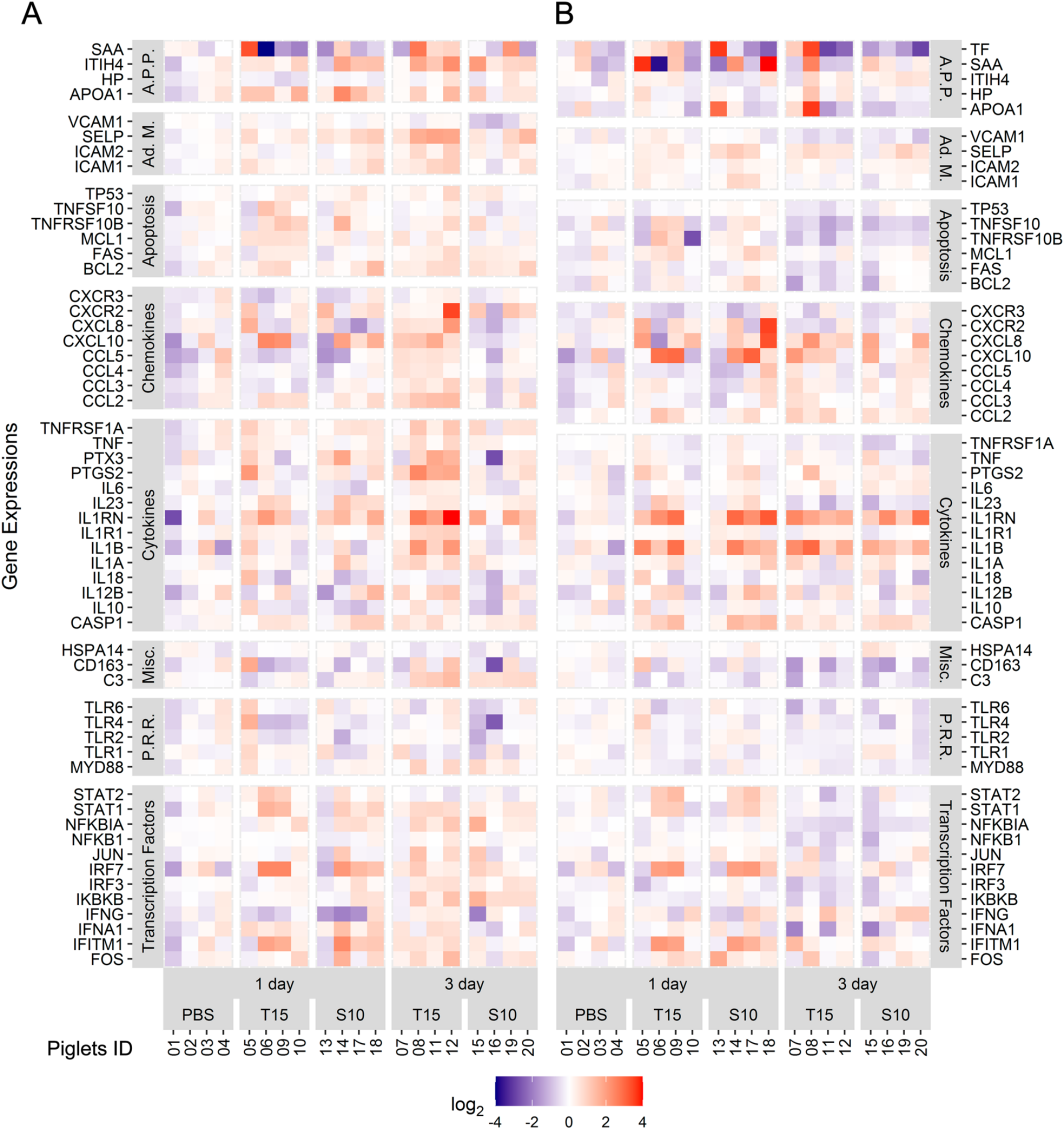
**Gene expression in submandibular and tracheobronchial lymph nodes after** ***S. suis*** **intranasal inoculation.** Samples from the submandibular (A) and tracheobronchial (B) lymph nodes were collected at 1 and 3 days after the intranasal inoculation of *S. suis* T15 (non-virulent) and S10 (virulent). Results at 1 day post-inoculation from piglets inoculated with PBS are also included as control. All the genes found to be quantifiable are shown irrespectively of their statistical significance. Gene expression was normalized relative to the PBS group and log_2_ transformed. Values are presented as a heat map. Numbers in abscissa axis represent animal ID. Color scale was limited to ± 4 and out of bounds values displayed with the maximum intensity color. Gene functional groups: A.P.P.: Acute Phase Proteins; Ad. M.: Adhesion Molecules; Misc.: Miscellaneous; P.R.R.: Pattern Recognition Receptors.

In contrast to the observations in the nasal samples, gene expression in the submandibular lymph node was generally less affected with fewer and smaller changes and only by the T15 strain (at 3 dpi), with no significant modulation of gene expression observed in animals inoculated with S10 strain at 1 or 3 dpi (Figure 5). At day 3 after the challenge with the non-virulent T15 strain, genes *IL1B* and *PTGS2* (indicative of inflammation) and *CCL2* and *SELP* (involved in recruitment of immune cells) were significantly >2-fold up-regulated vs*.* PBS control (Figure 5). In the case of *PTGS2* and *CCL2*, significant differences were also found between T15 and S10 inoculated animals, with up-regulation only by the non-virulent T15 strain. In the tracheobronchial lymph node, significant up-regulation was only observed for *CASP1* at 1 dpi in S10 inoculated pigs (Figure 5). Genes with significant changes lower than 2-fold when compared vs. the PBS group for both lymph nodes are included in Additional file 4. In addition, individual expression changes in submandibular and tracheobronchial lymph nodes are presented in Figure 6. Although some changes were observed in individual piglets, the response showed high variation within the groups and no statistical differences were found in our model (Figure 6 and Additional file 5). As an example, *IL1RN* in tracheobronchial lymph node: mean ± standard deviation of 3.92 ± 2.78 and 3.79 ± 1.18 for T15 strain at 1 and 3 dpi respectively; and 5.88 ± 3.49 and 4.06 ± 2.44 for S10 strain at 1 and 3 dpi respectively (all tissues values are available in Additional file 5).

In trachea, *IL10* and *TLR6* were significantly down-regulated in response to both strains, which coincided with a general trend towards down-regulation of the majority of genes examined in this tissue (Figure 5, Additional file 6). In lungs, more changes were detected in the caudal than in the cranial lobe, which only showed minor changes in *TLR6* in the S10 inoculated group (Additional file 4). In the caudal lobe, significant regulation with a >2-fold change was observed for *CXCL8* (IL8) (down-regulated by T15 at 1 dpi), *SAA* (up-regulated by T15 at 3 dpi), and *TNF* (down-regulated by T15 at 3 dpi, and by S10 at 1 and 3 dpi) compared to the PBS group (Figure 5). When comparing the responses to the two strains, *SAA* (>2-fold, Figure 5) and *TLR2* (<2-fold, Additional file 4) were significantly higher for the non-virulent T15 than the virulent S10 at 3 dpi. A few other significantly different (*P* < 0.05) <2-fold changes compared to the PBS group, between time points for the same strains, or between strains at the same time point, are shown in Additional file 4.

**Figure 7:**
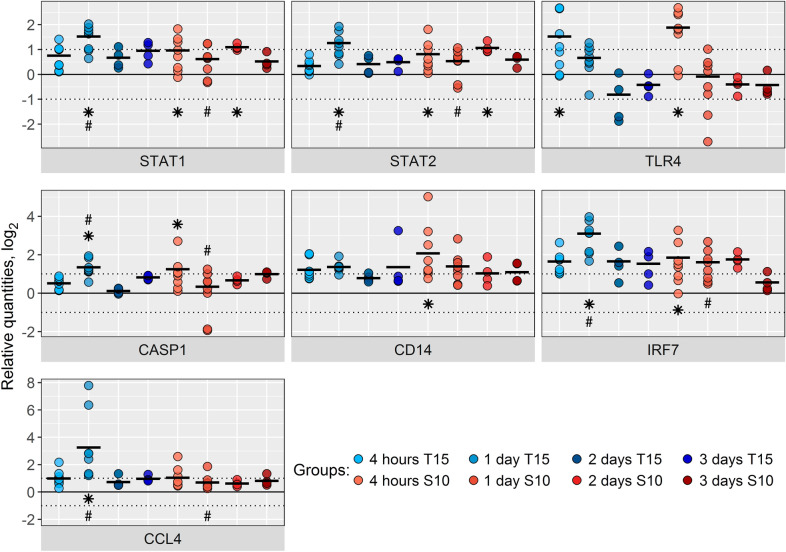
**Relative gene expression in blood after** ***S. suis*** **intranasal inoculation.** Log_2_ of the individual values and mean (black bars) of the relative gene expression in blood in *S. suis* inoculated piglets (gene expression was normalized relative to the 3 days before challenge mean for each inoculated group). Piglets were intranasally inoculated with *S. suis* T15 (non-virulent, blue bars) or S10 (virulent, red bars), and blood samples were taken at 4 h and 1, 2, and 3 days after inoculation. Genes with at least 1 significant difference in pairwise analysis (*P* < 0.05) when comparing different time points in the *S. suis* inoculated groups or between strains at the same time point, and with a mean greater than 2-fold change (log_2_ = 1) are shown. * indicates significant differences (*P* < 0.05) versus their respective 3 days before challenge time point. Differences between strains at the same time point are labelled with # (*P* < 0.05). *n* = 8 for both strains at 4 h post-infection and 1 dpi, *n* = 4 for both strains at 2 and 3 dpi. All expression values and significant differences can be found in Additional file 7.

**Figure 8:**
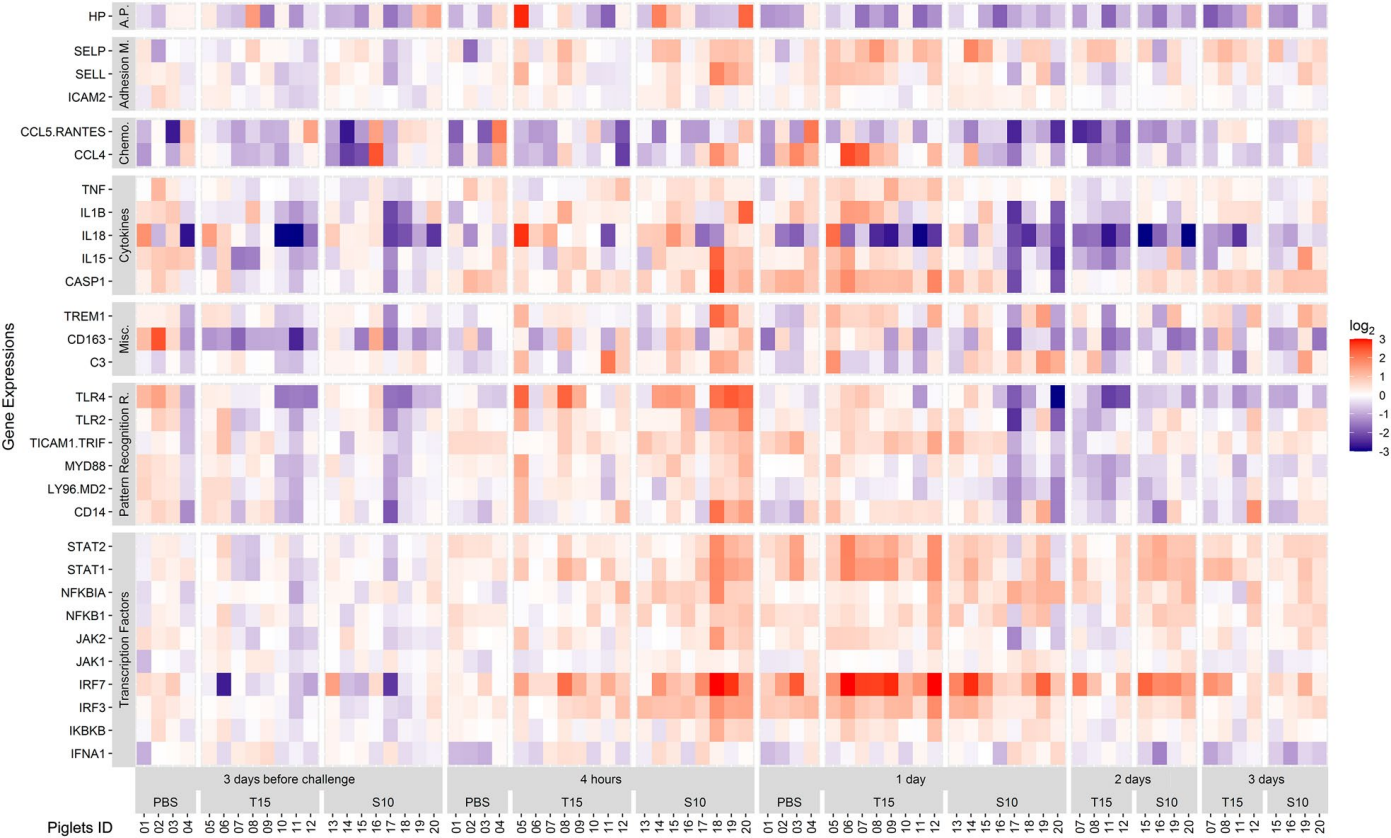
**Gene expression in blood after** ***S. suis*** **intranasal inoculation.** Samples were taken at 3 days before, and 4 h, 1, 2, and 3 days after the intranasal inoculation of *S. suis* T15 (non-virulent) and S10 (virulent). A group of piglets inoculated with PBS are also shown and served as control. All genes found to be quantifiable are shown irrespectively of their statistical significance. Gene expression was normalized relative to the 3 days before challenge mean for each inoculated group and log_2_ transformed. Values are presented as a heat map. Numbers in abscissa axis represent animal ID. Color scale was limited to ± 3 and out of bounds values displayed with the maximum intensity color. Gene functional groups: A.P.: Acute Phase Proteins; Adhesion M.: Adhesion Molecules; Chemo.: Chemokines; Misc.: Miscellaneous; Pattern Recognition R.: Pattern Recognition Receptors.

Analysis of whole blood indicated changes in gene expression patterns in response to the challenge with both *S. suis* strains, although the majority of changes were below 2-fold, with subtle differences in the temporal dynamics depending on the challenge strain (Figures 7 and 8, Additional file 7). The blood response to strain S10 was rapid with six genes showing >2-fold up-regulation at 4 h after challenge (*CASP1*, *CD14*, *IRF7*, *STAT1*, *STAT2*, and *TLR4*), and maintaining this difference at 2 dpi for *STAT1* and *STAT2* (Figure 7, Additional file 7). The response to the non-virulent strain T15 was more delayed, with a peak in the number of significantly up-regulated genes with a >2-fold change on 1 dpi (five genes, *CASP1*, *CCL4*, *IRF7*, *STAT1*, and *STAT2*), and only one gene (*TLR4*) significantly up-regulated at 4 h after the challenge (Figure 7, Additional file 7). The different response observed to both strains was statistically different only at 1 dpi for the following genes: *CASP1*, *CCL4*, *IRF7*, *STAT1*, and *STAT2*, with higher values in animals challenged with the non-virulent strain T15 (Figure 7, Additional file 7). Significant differences lower than 2-fold between strains were observed only at 1 dpi for the genes *IL1B*, *JAK2*, *TICAM1, TRIF*, and *TNF*, with higher values for T15 than for the S10, and also for *NFKBIA*, but in the opposite direction (Additional file 7).

Consistent with the absence of systemic disease and/or overt systemic reactions to the intranasal *S. suis* inoculation, very few genes were significantly affected in liver and spleen (Additional file 8). Despite the small magnitude of these changes (all with <2-fold changes), some statistical differences between strains were observed in liver at 1 dpi, with lower values in piglets inoculated with the virulent strain S10 for *BCL2*, *TNFRSF1A*, and *TP53*. Individual values for all the genes analyzed in these tissues are presented as heat maps in Additional file 9.

## Discussion

Pathogens use different mechanisms to evade the innate immune system, the first line of defense against them, and to colonize the host. In *S. suis* infection, the host’s immune response combined with the virulence of the infecting strain play important roles in achieving colonization and, subsequently, in the possible development of the disease [30].

Although it is difficult to reproduce disease with this bacterium using the intranasal route of inoculation, it has been used on numerous occasions to study host–pathogen interactions [30]. In the present study, despite the fact that systemic disease did not develop in the inoculated animals in the short course of the study, we did observe various host responses (including fever) induced by strains S10 and T15, with different known virulence potential, during the first steps of infection. For most of the sample types examined in this work, the limited number of animals and high individual variations made it difficult to correlate a clear gene expression pattern or inflammatory marker consistently with the virulence of the strain. However, for the nasal mucosa the transcriptional response did in fact reflect the virulence potential of the inoculated *S. suis* strain. Despite these limitations, this study used an experimental model that reproduces *S. suis* natural infection of pigs, providing for the first time a comprehensive overview of the host innate immune response induced by *S. suis* during upper respiratory tract colonization. In addition, the present study paves the way for more extensive mechanistic studies on modulation of host immunity by this important swine pathogen.

Interestingly, both strains induced an early pro-inflammatory response locally in the nasal mucosa; however, the return to baseline gene expression levels was faster for the non-virulent strain (T15). Among genes up-regulated by both strains at the nasal mucosa, *IL1B* is a cytokine that acts as a master regulator of inflammation by controlling a variety of innate immune processes [31]. Several studies have reported the capacity of *S. suis* to induce IL-1 cytokine family members by a variety of cell types [32, 33]. In addition to *IL1B*, up-regulation of the interferon-regulatory factor 1 (*IRF1*) suggests activation of the interferon (IFN) pathway during *S. suis* colonization, including expression of *CXCL10*, a chemokine gene that can be up-regulated in response to IFN-γ/IRF1 signaling pathway [34]. The IFN pathways can play either a regulatory or a pathological role depending on the virulence of the *S. suis* strain or the specific clinical manifestation of the disease, as previously suggested [33, 35].

In the submandibular lymph node, gene expression related to recruitment of immune cells (such as expression of the chemokine *CCL2* and the adhesion molecule P selectin encoded by *SELP*) and to inflammation (*IL1B*) was mainly observed after colonization with the non-virulent strain. These seemingly contradictory results observed between nasal mucosa and the submandibular lymph node may reflect intrinsic properties of the strains, with different molecular composition, including the presence of virulence-associated proteins in the S10 strain (e.g., the Muramidase-Released Protein [MRP] or the extracellular factor [EF] protein) that are absent in T15 [24].

In the absence of clinical manifestations and histopathological lesions, the observed modulation of the innate immune response by *S. suis* colonization could be considered a homeostasis-restoring state of inflammation [36], which is considered different from pathological inflammation. It has been suggested that such state may be maintained by pattern recognition receptors (PRRs) expressed in stromal and/or immune cells, detecting endogenous ligands and/or pathogens [36]. In agreement with this concept, expression of interleukin-1 receptor antagonist (IL-1RA; encoded by *IL1RN*) was observed in nasal samples and that of the enzyme cyclooxygenase-2 (COX-2), encoded by *PTGS2*, was found in submandibular lymph node (and mainly induced by the non-virulent strain). COX-2-derived metabolites are important regulators of inflammation [37] and IL-1RA competitively inhibits IL-1 binding to cell-surface receptors. Maintenance of a balance between IL-1 and IL-1RA is important in preventing the development or progression of inflammatory disease [38]. It has been suggested in other models that selective induction of IL-1RA might facilitate mucosal colonization by bacteria. IL-1RA also plays a critical role in maintaining a homeostatic and balanced microbiota [39, 40]. Further studies are required to delineate the link between *S. suis* colonization and the induction of a homeostasis-restoring state of inflammation, including a potential regulatory role of IL-1RA and/or COX-2.

The systemic response was limited as no clinical invasive disease was observed and *S. suis* was not found in blood. The observed minor changes in gene expression in systemic samples could be a consequence of the ongoing local response at the upper respiratory track. Indeed, up-regulated genes were associated to the IL-1 or the IFN pathways (such as *CASP1*, *IRFs*, and *STATs*), which seem to predominate during the innate immune response induced by *S. suis* colonization. However, massive activation of these and other pro-inflammatory pathways (cytokine storm) are known to be involved in pathological inflammation during *S. suis* systemic disease leading to septic shock [41]. Nevertheless, the transcriptional patterns in blood showed that the host response to the virulent challenge was rapid, peaking within hours after challenge, which coincided with elevated body temperature. In contrast, the circulating response to the non-virulent challenge was more delayed and did not coincide with the fever response. This correlation between up-regulation of pro-inflammatory cytokines and fever agrees with the initial course of disease in other pig infection models [42]. Internal organs such as liver, spleen, kidney, or heart, are invaded after *S. suis* reaches systemic circulation [43]. However, in the present study, the piglets did not develop systemic disease and, accordingly, the splenic and hepatic response to both *S. suis* strains showed a low number of genes significantly affected and with low magnitude. This lack of systemic disease may be explained by the route of inoculation, intranasal, unaided by acetic acid or viral co-infection [19, 20], the short time of the study, or the ability of the host to control the infection before bacteria could reach the bloodstream.

Transcriptional results from trachea and lungs indicate that the host response or bacterial spread beyond the nasal cavity and further down the respiratory tract for the duration of the experiment was limited. Specific serotype detection by IHC was achieved in the tissues in which more mucus remained after the paraffin treatment, like the alveolar sac in lungs or the characteristic sinuous tissue of the cribriform plate of the ethmoid bone. In other respiratory tissues, bacteria were only detected in mucus or a few of them attached to the epithelium, which is consistent with the sub-clinical infection and the low response observed in the trachea. Colonization thus appears to primarily affect the host response locally at the site of colonization, with little or no widely disseminated response beyond the nasal cavity. Regarding the localization of *S. suis* S10 detected in the cribriform plate of the ethmoid, it cannot be ruled out that this site may serve as a non-hematogenous route to the central nervous system. This route has previously been suggested for *Streptococcus pneumoniae* [44] and demonstrated for others bacteria such as *Neisseria meningitidis* and *Burkholderia pseudomallei* [45, 46], as well as for ameboflagellates (*Naegleria fowleri*; [47]) or viruses (SARS-CoV-2; [48]). This hypothesis deserves further analysis.

This study provides information for understanding the colonization of *S. suis* (first step of infection) and the potential mechanisms involved in the early local innate immune response, which might either favor colonization without disease development or rather colonization followed by systemic invasion. Our results seem to reflect a host response to this non-virulent *S. suis*, which is characterized by rapid control at the site of inoculation, probably mediated by a sustained immune response at the associated lymph node. In contrast, the virulent strain used seem to prevent a robust lymph node response, and, in consequence, they are maintained at the site of inoculation, where they continue to elicit inflammatory mediators. Several factors might dictate these outcomes, including host and environment factors, as well as the virulence potential of the strain.

## Supplementary Information

**Additional file 1.** **RNA quality.** Mean values with the minimum and maximum range for RNA concentration and qualities for each sample type.

**Additional file 2. Primer sequences and amplification efficiency.** List of primers used, including primer sequences, amplicon length and amplification efficiency by tissue.

**Additional file 3. Gene expression in nasal samples.** Gene expression from nasal swabs samples, including means, standard deviations, and *P*-values.

**Additional file 4. Significant gene expression with < 2-fold changes in lymph nodes, trachea and lungs after** ***S. suis*** **intranasal inoculation.** Log_2_ of the individual values and mean (black bars) of the relative gene expression in different tissues of *S. suis* inoculated piglets. Piglets were intranasally inoculated with *S. suis* T15 (non-virulent, blue bars) or S10 (virulent, red bars), and necropsies were performed at 1 and 3 days post-infection. Gene expression was normalized relative to the PBS group. The values and means are shown for the indicated groups (challenge strain and time point) having at least one significant difference when compared to the PBS group and with a mean lower than 2-fold change (log_2_ = 1). LN Trbr: Tracheobronchial lymph node; Lung Cr: Lung Cranial; Lung Cd: Lung Caudal. * indicates significant differences (*P* < 0.05) versus the PBS group. Differences between strains at the same time point are labelled with # and differences between time points for the same strain are labelled with +, *P* < 0.05, in both cases. *n* = 4 for each group. All expression values and significant differences can be found in Additional file 5.

**Additional file 5. Gene expression in tissues samples.** Gene expression from tissues samples, including means, standard deviations, and *P*-values.

**Additional file 6. Gene expression in different respiratory tissues after** ***S. suis*** **intranasal inoculation.** Samples from trachea (A), caudal lung (B), and cranial lung (C) were collected at 1 and 3 days after the intranasal inoculation of *S. suis* T15 (non-virulent) and S10 (virulent). Results at 1 day post-inoculation from piglets inoculated with PBS are also included as control. All the genes found to be quantifiable are shown irrespectively of their statistical significance. Gene expression was normalized relative to the PBS group and log_2_ transformed. Values are presented as a heat map. Numbers in abscissa axis represent animal ID. Color scale was limited to ± 4 and out of bounds values displayed with the maximum intensity color. Gene functional groups: Acute P.P.: Acute Phase Proteins; Chemo.: Chemokines; P.R.: Pattern Recognition Receptors.

**Additional file 7. Gene expression in blood.** Gene expression from blood samples, including means, standard deviations, and *P*-values.

**Additional file 8. Significant gene expression in spleen and liver after** ***S. suis*** **intranasal inoculation.** Log_2_ of the individual values and mean (black bars) of the relative gene expression in spleen and liver in *S. suis* inoculated piglets. Piglets were intranasally inoculated with *S. suis* T15 (non-virulent, blue bars) or S10 (virulent, red bars), and necropsies were performed at 1 and 3 days post-infection. Gene expression was normalized relative to the PBS group. The values and means are shown for the indicated groups (challenge strain and time point) having at least one significant difference when compared to the PBS group. * indicates significant differences (*P* < 0.05) versus the PBS group. Differences between strains at the same time point are labelled with # and differences between time points for the same strain are labelled with +, *P* < 0.05, in both cases. *n* = 4 for each group. All expression values and significant differences can be found in Additional file 5.

**Additional file 9.** **Gene expression in spleen and liver after** ***S. suis*** **intranasal inoculation.** Samples from spleen (A) and liver (B) were collected at 1 and 3 days after the intranasal inoculation of *S. suis* T15 (non-virulent) and S10 (virulent). Results at 1 day post-inoculation from piglets inoculated with PBS are also included as control. All the genes found to be quantifiable are shown irrespectively of their statistical significance. Gene expression was normalized relative to the PBS group and log_2_ transformed. Values are presented as a heat map. Numbers in abscissa axis represent animal ID. Color scale was limited to ± 3 and out of bounds values displayed with the maximum intensity color. Gene functional groups: A.P.P. and Acute P.P.: Acute Phase Proteins; Ad. M.: Adhesion Molecules; Chemo.: Chemokines; Misc.: Miscellaneous; P.R.R.: Pattern Recognition Receptors; Trans. F.: Transcription Factors.

## Data Availability

All data generated or analysed during this study are included in this published article (and its additional files).
